# Using gEUD based plan analysis method to evaluate proton vs. photon plans for lung cancer radiation therapy

**DOI:** 10.1002/acm2.12281

**Published:** 2018-02-13

**Authors:** Zhiyan Xiao, Wei J Zou, Ting Chen, Ning J Yue, Salma K Jabbour, Rahul Parikh, Miao Zhang

**Affiliations:** ^1^ Department of Radiation Oncology Robert Wood Johnson University Hospital The Cancer Institution of New Jersey‐Rutgers University New Brunswick NJ USA; ^2^ Department of Radiation Oncology University of Pennsylvania Philadelphia PA USA

**Keywords:** gEUD, lung cancer, proton therapy

## Abstract

The goal of this study was to exam the efficacy of current DVH based clinical guidelines draw from photon experience for lung cancer radiation therapy on proton therapy. Comparison proton plans and IMRT plans were generated for 10 lung patients treated in our proton facility. A gEUD based plan evaluation method was developed for plan evaluation. This evaluation method used normal lung gEUD(a) curve in which the model parameter “a” was sampled from the literature reported value. For all patients, the proton plans delivered lower normal lung V_5 Gy_ with similar V_20 Gy_ and similar target coverage. Based on current clinical guidelines, proton plans were ranked superior to IMRT plans for all 10 patients. However, the proton and IMRT normal lung gEUD(a) curves crossed for 8 patients within the tested range of “a”, which means there was a possibility that proton plan would be worse than IMRT plan for lung sparing. A concept of deficiency index (DI) was introduced to quantify the probability of proton plans doing worse than IMRT plans. By applying threshold on DI, four patients’ proton plan was ranked inferior to the IMRT plan. Meanwhile if a threshold to the location of curve crossing was applied, 6 patients’ proton plan was ranked inferior to the IMRT plan. The contradictory ranking results between the current clinical guidelines and the gEUD(a) curve analysis demonstrated there is potential pitfalls by applying photon experience directly to the proton world. A comprehensive plan evaluation based on radio‐biological models should be carried out to decide if a lung patient would really be benefit from proton therapy.

## 1 INTRODUCTION

Lung cancer is the leading cause of cancer death in the world.^1^ It is anticipated that the incidence and mortality will continue to increase worldwide because of smoking, environmental pollution, and an aging population. Lung cancer radiation therapy is highly efficacious particularly for patients with disease limited to the thorax, but carries the risk of significant morbidities particularly radiation induced pneumonitis.^2^, ^3^ Most often, the major challenge in planning lung radiation therapy is reducing irradiation to the normal lung parenchyma. The current clinical experience based on photon radiation therapy shows low dose to large amount of normal lung is a strong indicator of radiation introduced lung damages as well as the V_20 Gy_.^3^, ^4^

As a charged particle, proton has a finite range in the patient. With careful planning, normal tissue distal to the target can be spared. Planning studies comparing proton vs. photon plans for lung cancer treatment demonstrated proton plans score lower number on the mean dose, the V_20 Gy_, and the V_5 Gy_ for normal lung.^4^, ^5^ Based on the current clinical guidelines, it would appear that proton would reduce radiation toxicities compared to a matched photon plan.^6^, ^7^, ^8^ More specifically, based on dose volume histogram (DVH) constraints specified in current clinical guidelines, the proton would be superior to its photon peer. However, it is not understood whether the direct transfer of knowledge from photon world to proton world is valid? Without any clinical trials, the judgment is hard to draw. From physics point of view, a proton plan would be superior to a photon plan if its normal lung DVH curve were reduced compared to the photon plan with similar target coverage. For any other cases, when DVH curves from a proton plan and photon crossed at certain point, a clear ranking would be difficult. Apparently, the proton plans could not always follow the mentioned criteria for all patients. Therefore, it is important to validate, if is not to introduce new, any clinical knowledge we draw from the photon world to apply for lung cancer proton radiation therapy.

Comparing to DVH, generalized Equivalent Uniform Dose (gEUD) is a single value summarizing the DVH curve information to represent the biological effect of a 3D dose distribution to an organ.^9^, ^10^ The calculation follows an easy formula with a biological endpoint specific parameter “a”. Although gEUD is easy to calculate and to compare, its strong dependence on the selection of “a” value makes it challenging for plan comparison. In this study, we proposed a gEUD based plan ranking mechanism. We design it to be a subjective plan ranking tool by considering the probability range of the “a” value. With this gEUD based plan ranking method in hand, we are about to approve the hypothesis that a lung proton plan with better DVH points according to current clinical guidelines may not be a safer plan than a photon intensity modulated radiation therapy (IMRT) plan.

## 2 METHODS

Ten non‐small cell lung cancer (NSCLC) patients previously treated with proton in our institution were included in this study. All the patients were simulated with 4DCT scan. They all had gross tumor motion less than 10 mm throughout the breathing period to be qualified for proton treatment. The internal target volume (ITV) and critical organs, including heart, spinal cord, and normal lung, were contoured by a certified oncologist. The ITV was delineated from the maximum intensity projection (MIP) image from 4DCT which encompassing the gross tumor moving area and micro‐extension of the disease. In photon plans, the target was the planning target volume (PTV) which was generated with 5 mm uniform expansion from the internal target volume (ITV) to count for the setup uncertainty. In proton plans, the target was defined as the ITV along. For each proton beam the aperture was generated with 10 mm lateral expansion from the ITV to count for the setup uncertainty and beam penumbra. Along the beam direction, both the proton range uncertainty and the treatment setup uncertainty would affect the target coverage. The distal and proximal margin from the ITV was calculated as the square root value of the range uncertainty and the setup uncertainty assuming those two factors were independent from each other. Based on multiple institutions’ experience,^11^, ^12^ 3 mm plus 3.5% range uncertainty value for proton beam was used in our institution. The compensator smearing was 10 mm.

Eclipse V11.5 (Varian, Palo Alto, CA, USA) treatment planning system was used for planning. Photon IMRT plans and proton double scattering plans were generated with a Varian 21EX machine and a Mevion S250 double scattering proton therapy system (Mevion, Littleton, MA, USA) commissioned in Eclipse. The prescription dose was 6000 cGy in 30 fractions. The proton dose was converted to cobalt dose equivalent using the current clinical standard relative biological effectiveness (RBE) of 1.1.^13^ The photon IMRT plan used five fields beam arrangement. In proton plans, 2–3 equally weighted fields separated by 30° were used with each field covering the whole target. Both plans were planned to have the same target coverage while dose to normal tissue was minimized to its own capacity. The photon IMRT plans were optimized to have 95% of the PTV covered by the 95% of the prescription dose. In the proton plans the plan was renormalized to ensure 98% of the ITV covered by 98% of the prescription dose. The final plans were evaluated by the same oncologist.

Maximum cord dose, mean heart dose, mean lung dose, lung V_5 Gy_, and V_20 Gy_ were extracted from each plan for comparison. Differential dose‐volume‐histogram (DVH) was calculated for individual organ and exported for gEUD calculation.

gEUD for normal lung was calculated following eq. (1) for both proton and photon plans. 10

(1) $$gEUD={\left(\sum_{i}v_{i}\times D_{i}^{a}\right)}^{\frac{1}{a}}$$

*v*_*i*_ is fractional volume, *D*_*i*_ is dose bin and the *a* value is an organ and endpoint‐specific value that takes into account the organ's response to inhomogeneous dose. The absolute value of gEUD strongly depends on the choosing of parameter “*a*”. During computation, a larger “*a*” gives higher weight to hot spots in the DVH, and a smaller “*a*” would weigh more on a lower dose to a large volume. Clinical studies reported that the “*a*” value for normal lung falls between 0.6 and 3. 14, 15, 16, 17, 18 With such large range, it is impossible to pick a specific value to calculate normal lung gEUD and rank plans deterministically.

The gEUD based plan evaluation method we developed during this study is as follow: first, it was carried out by calculating the normal lung gEUD from a given plan using “a” values between 0.6 and 3. Then, the curve of gEUD vs. “a” for that plan was plotted. If plan B's gEUD curve was always lower than plan A's within the range of all possible “a” values, then plan B was ranked better than plan A, and vice versa. On the other hand, if the gEUD curves between two plans crossed within the range of tested “a”s, then a clear ranking would be difficult. In that case, a deficiency index (DI) summarizing the overall gEUD differences between plan A and B across the range of possible “a”s was calculated as follow:

(2) $$\begin{array}{cc} & DI=A/B\\ & A=\sum_{gEUD_{\mathrm{proton}}\left(a\right)>gEUD_{\mathrm{photon}}\left(a\right)}\left[gEUD_{\mathrm{proton}}\left(a\right)-gEUD_{\mathrm{photon}}\left(a\right)\right]/gEUD_{\mathrm{photon}}\left(a\right)\\ & B=\sum_{gEUD_{\mathrm{proton}}\left(a\right)<gEUD_{\mathrm{photon}}\left(a\right)}\left[gEUD_{\mathrm{photon}}\left(a\right)-gEUD_{\mathrm{proton}}\left(a\right)\right]/gEUD_{\mathrm{photon}}\left(a\right) \end{array}$$

In another word, A is the area gEUD_proton_(a) larger than gEUDphoton (a) and B is vice versa. A larger DI shows a proton plan would likely to give higher dose to normal lung than its photon peer. In a case gEUD_proton_(a) were constantly lower than gEUD_photon_(a), the DI would be zero which means the proton plan would be certainly superior to the photon plan for lung sparing. Other than DI, the point where two gEUD curves crossed (a_crossing_) was also recorded for analysis.

## 3 RESULTS

**Figure 1:**
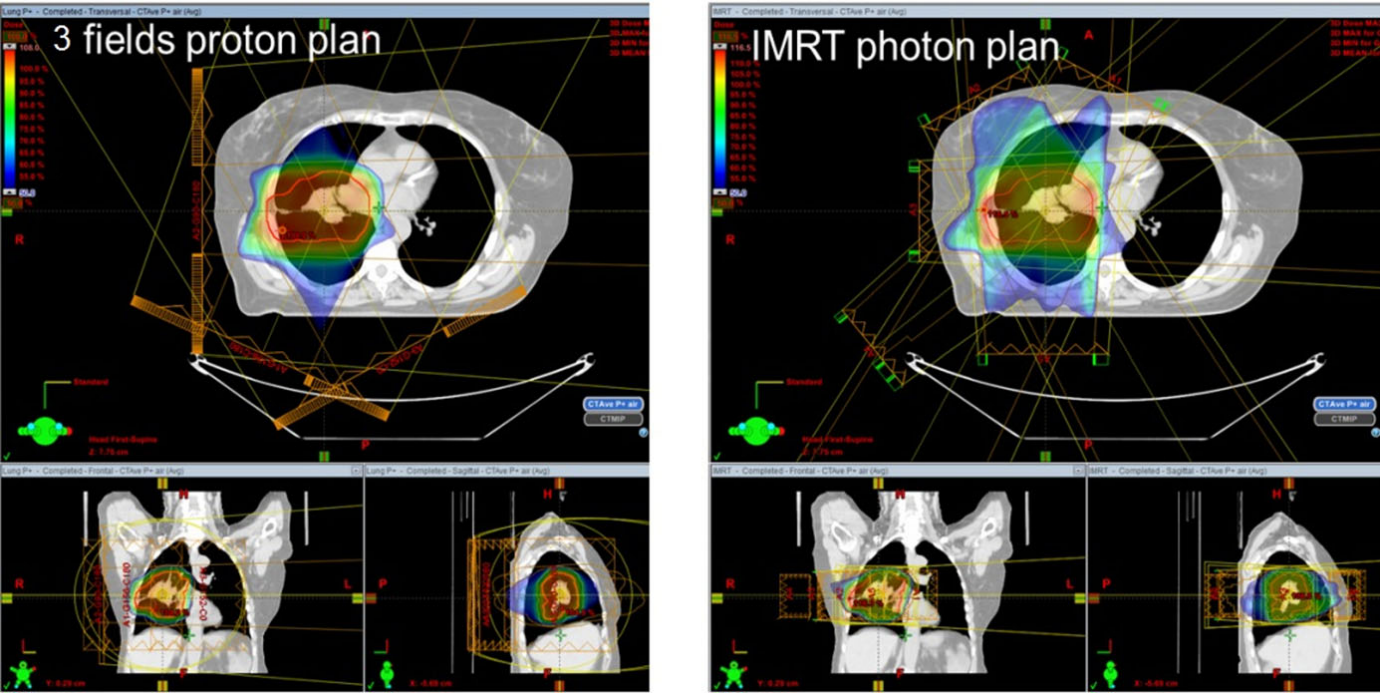
Dose distribution of a proton plan (left) and its peer photon IMRT plan (right).

A typical dose distribution from a proton plan and a photon IMRT plan is shown in Fig. 1. For all patients, proton plans yielded similar PTV coverage and lower dose to heart and spinal cord than the comparing photon plans. The results are summarized in Table 1. For normal lung, proton plans yielded lower V_5 Gy_ but comparable V_20 Gy_ to their photon peers. On average, normal lung V_5 Gy_proton_ was 34.5% than 58.9% for the V_5Gy_photon_.

**Table 1:** Dosimetric value comparison between proton plans and IMRT plans for all tested cases. The difference highlighted in bold font was calculated as Value_proton_ – Value_IMRT_

Case no.	Tx Tech	ITV	Cord	Heart	Normal lung		
		Vpx (%)	Max (cGy)	Mean (cGy)	Mean (cGy)	V_5 Gy_ (%)	V_20 Gy_ (%)
1	IMRT	90.0	1081.0	2191.1	1429.0	57.7	25.6
	Proton	92.1	133.4	1181.8	1194.8	38.0	30.4
	**Diff.**	**2.1**	**−947.6**	**−1009.3**	**−234.3**	**−19.8**	**4.8**
2	IMRT	90.0	208.3	483.7	674.3	46.6	6.2
	Proton	94.6	1.9	337.9	542.1	29.9	10.9
	**Diff.**	**4.6**	**−206.4**	**−145.8**	**−132.2**	**−16.7**	**4.7**
3	IMRT	92.8	802.4	4836.9	931.7	64.6	9.4
	Proton	93.2	0.2	3866.4	513.7	23.0	8.2
	**Diff.**	**0.4**	**−802.2**	**−970.5**	**−418.0**	**−41.6**	**−1.2**
4	IMRT	90.1	418.9	1639.6	1234.7	65.3	22.6
	Proton	98.2	0.3	1030.3	1015.6	30.1	21.1
	**Diff.**	**8.1**	**−418.6**	**−609.3**	**−219.1**	**−35.1**	**−1.5**
5	IMRT	90.2	1054.5	113.5	705.2	34.0	12.2
	Proton	94.8	834.0	89.1	664.3	16.7	12.9
	**Diff.**	**4.6**	**−220.5**	**−24.4**	**−40.9**	**−17.3**	**0.7**
6	IMRT	95	970.6	657.3	1388.9	49.0	25.9
	Proton	93.1	443.3	968.1	1491.4	42.2	28.8
	**Diff.**	**−1.9**	**−527.3**	**310.8**	**102.5**	**−6.8**	**2.9**
7	IMRT	90.0	1287.6	1424.7	1613.0	66.1	34.5
	Proton	94.5	420.8	1552.4	1636.2	50.3	34.8
	**Diff.**	**4.5**	**−866.8**	**127.7**	**23.2**	**−15.8**	**0.3**
8	IMRT	89.9	1887.9	1177.5	1335.4	56.6	27.0
	Proton	90.0	923.9	952.1	1245.0	38.6	26.2
	**Diff.**	**0.1**	**−964.0**	**−225.4**	**−90.4**	**−18.0**	**−0.8**
9	IMRT	92.2	1251.3	1902.1	1555.8	72.7	25.8
	Proton	92.1	589.5	1064.4	1288.1	39.1	22.8
	**Diff.**	**−0.1**	**−661.8**	**−837.7**	**−267.7**	**−33.6**	**−2.9**
10	IMRT	95.3	659.8	1474.9	1913.7	75.9	33.6
	Proton	90.0	315.8	529.6	1301.1	37.2	29.6
	**Diff.**	**−5.3**	**−344.0**	**−945.3**	**−612.6**	**−38.8**	**−3.9**

**Figure 2:**
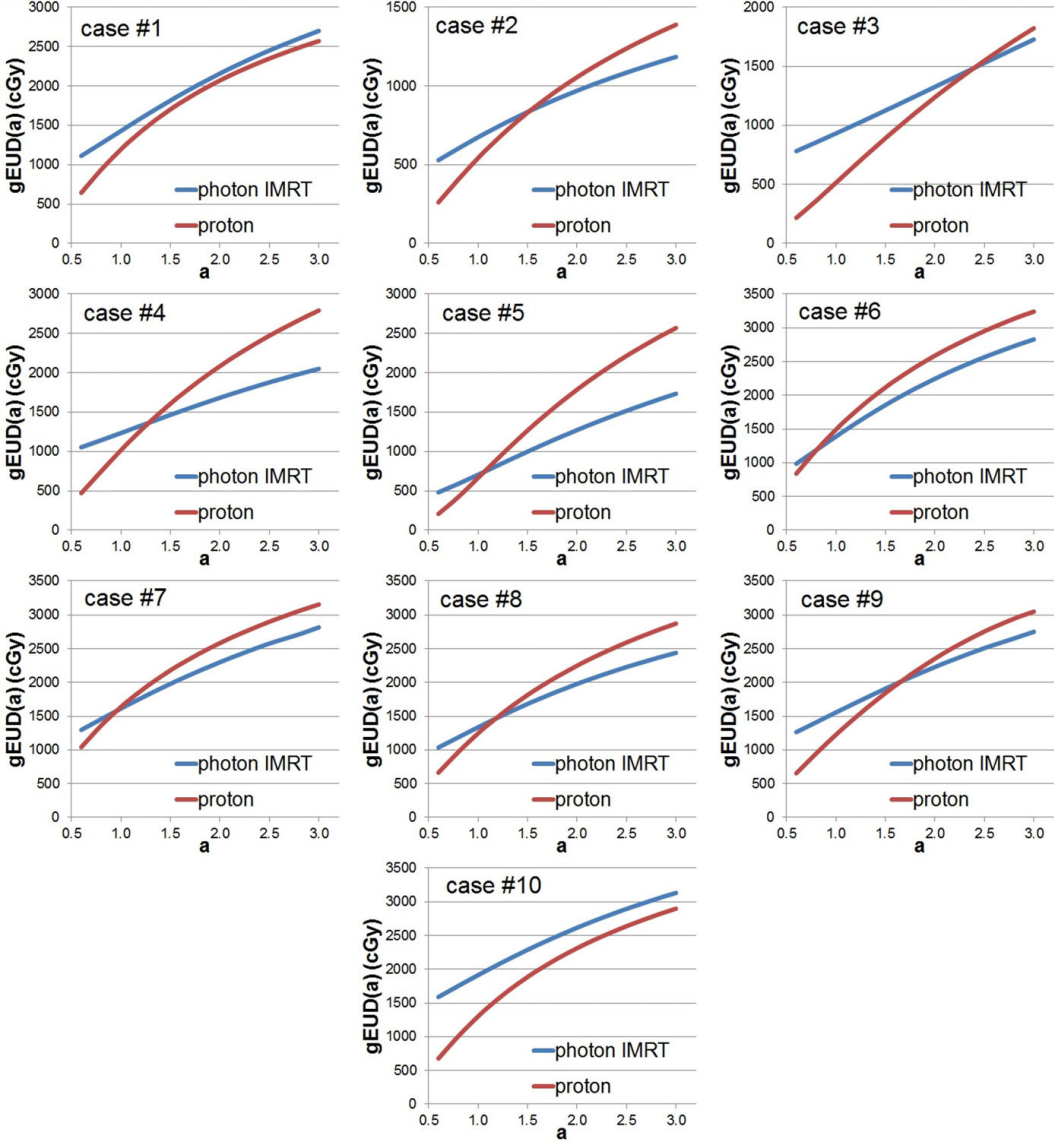
gEUD(a) curve comparison between proton and photon IMRT plans for all tested cases.

Normal lung gEUD versus “a” values are plotted in Fig. 2. The gEUD value monotonically increased with increasing “a” for all proton and photon plans. For case #1 and #10 gEUD_proton_(a) were constantly lower than gEUD_photon_(a). Therefore, the proton plans were certainly superior to the photon plans for lung sparing. However, for the rest cases, gEUD_proton_(a) and gEUD_photon_(a) crossed at certain “a” value (a_crossing_). Table 2 summarizes the a_crossing_ and DI for each case. The DI ranged from 0.050 to 9.921 and a_crossing_ were between 0.8 and 2.5.

**Table 2:** The deficiency index (DI) and a_crossing_, “a” value when gEUD_proton_(a) and gEUD_photon_(a) curve crossed, for all tested cases. The bold font highlights cases with DI >1, and a_crossing_ <1.75, which are cases when proton plan was determined inferior to photon IMRT plan. “NA” in the a_crossing_ column shows curve crossing was not observed in the tested range

Case no.	DI	a_crossing_
1	0.000	NA
2	0.764	**1.5**
3	0.050	2.5
4	0.494	1.75
5	**4.041**	**1.1**
6	**9.921**	**0.8**
7	**4.324**	**1.0**
8	**2.005**	**1.3**
9	0.458	**1.6**
10	0.000	NA

## 4 DISCUSSION

In this study, the same parameter “a” was used to calculate gEUD value for both proton and photon plans. The “a” value reveals the radio‐biological response of cells in a given organ. Different types of radiation would result in different microscopic dose deposition which affects the cell response. Mathematically the dose between different radiations could be converted to cobalt equivalent dose using the RBE factor. With the same cobalt equivalent dose, a proton beam and a photon beam would result in the same biological end‐point. Therefore, the same “a” value for an organ could be shared between proton therapy and photon therapy. However, the current widely used proton RBE factor of 1.1 is very likely a simplification of the actual radio‐biological effectiveness of proton beam. Therefore, in the future clinical observations using proton therapy, different “a” values may be concluded. However, as for this study, using the same “a” factor for plan evaluation is a reasonable assumption based on current clinical standard.

Judging by photon‐based experience, proton plans were superior to photon IMRT plans for all compared cases. It resulted in lower mean dose, lower V_5Gy_, and lower V_20 Gy_ to normal lung while providing similar target coverage. However, based on gEUD(a) curve analysis, only in 2 out of 10 cases proton would be certainly better than photon for lung sparing. For the rest 8 cases, careful analysis based on current available radio‐biological evidence should be applied. It is a general believe that lung can be described more likely as a “parallel organ” in which the mean dose (a = 1) is more correlated to the radiation damage.^19^ However, published data^14^, ^15^, ^16^, ^17^, ^18^ also suggest there is a possibility of lung having more “serial organ” behavior with the “a” value larger than 1. For those eight undetermined cases, the proton plans would be inferior to the photon plans if lung has more “serial organ” behavior with the true “a” value higher than a_crossing_. One criterion we could use for plan evaluation would be applying a threshold on a_crossing_. The tested range of “a” value of [0.6, 3] was from literature including various confidence intervals (CI) determined by each report. The details of each study and their findings are summarized in Table 3. Among those studies, Tucker et al.^16^ reported the maximums “a” value of 1.75. If we set the threshold by that, then for plans with a_crossing_ larger or equal to 1.75, the proton plan would be better than the photon plan, and vice versa. Following this ranking method, in four of 10 cases proton plans would result in lower lung toxicity than photon plans.

**Table 3:** The summary of each gEUD regression studies and their finds. In all the studies, the endpoint was radiation pneumonitis which requires either steroid or oxygen. CI stands for confidence interval

Studies	Year	Study size	a	95% CI
Bradley et al^14^	2007	324	0–1	–
Seppenwoolde et al^15^	2003	382	1.01	–
Tucker et al^16^	2008	576	1.75	0.76–3.85
Moiseenko et al^17^	2003	55	0.98	0.65–1.96
Liu et al^18^	2013	164	0.1–10	–

One pitfall of a_crossing_ thresholding method was the exclusion of potential variation in normal lung “a” value. To include that factor and also consider the severity when proton plans hypothetically doing worse than photon, the DI might be better for plan evaluation. If we assuming the normal lung “a” value has a uniform possibility within the range of tested “a”, DI = 1 might be used as the threshold. For DI larger or equal to 1, the proton plan would be worse than the photon plan, and vice versa. Following this ranking method, in six of 10 cases proton would be better than photon for lung sparing. Interestingly, the four cases determined by the a_crossing_ thresholding method are all included in the 6 cases determined by DI thresholding method.

Both a_crossing_ and DI thresholding methods are empirical evaluation tools based on currently available data. There are discrepancies between those evaluation methods. To accurately determine the plan quality, more reliable radio‐biological dataset is required. However, regardless of different approaches, both methods confirmed that the proton lung plan was not always better than the photon IMRT plan for lung sparing as determined by the current clinical guideline values.

**Figure 3:**
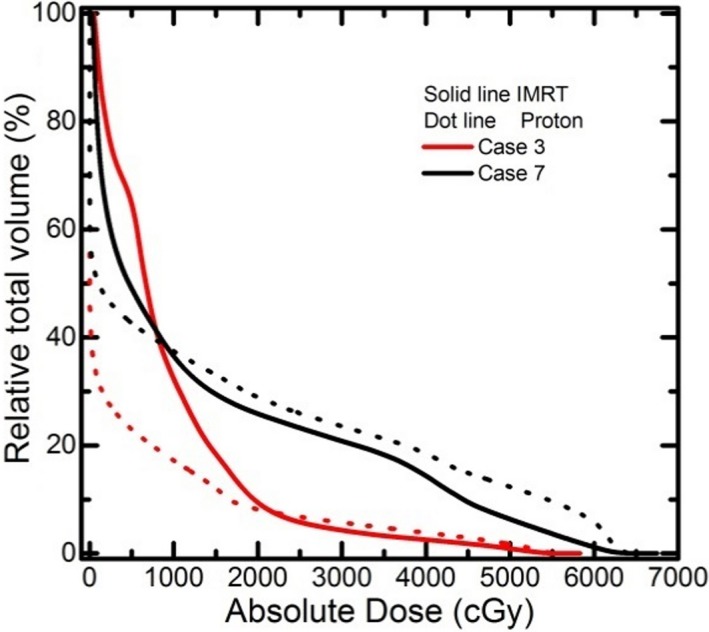
Comparison of normal lung DVH curves between proton and photon IMRT plan for selected 2 cases.

In this study, the double scattering proton deliver technique was compared with the photon IMRT. Without intensity modulation, proton plans naturally would not be as conformal as the IMRT plans. However, the study objective is not to compare the superiority of the delivery techniques other than examining the failure of using abstracted knowledge from one technique applying to the other. To answer the question why the compared proton double scattering plan would be worse than its photon peer for lung sparing, we compared the normal lung DVH from both plans. Figure 3 plots the normal lung DVH curves for case #3 (a_crossing_ = 2.5, DI = 0.050) and case #7(a_crossing_ = 1.0, DI = 4.324). The proton plan achieved smaller low dose region by sacrificing larger high dose volume to the normal lung than that from the photon plans. Comparing case #3 to #7, a proton plan with larger high dose volume to the normal lung than its photon peer (#7) would be worse than the one with smaller high dose volume to the normal lung (#3). Due to the incorporating of range uncertainty, a proton plan most likely would be less conformal to the target as a photon IMRT plan. Aggregated with complex target shape and the signature non‐conformal proximal dose distribution from the double scattering proton delivery, the dose conformality would be futher worse in certain cases than the others. The poor dose conformality translates to larger high dose volume to the normal lung in proton plans which result in higher lung dose based on gEUD analysis. With better management of the range uncertainty and using advanced delivery technique, e.g., pencil beam scanning, a more conformal dose distribution might be achievable with proton. In that case, intensity modulated proton therapy (IMPT) plans would be better than photon plans. 20 However, this investigation would exceed the scope of this paper and remains as a topic for future study.

## 5 CONCLUSIONS

In this study, we propose a comprehensive plan evaluation method based on gEUD(a) and the published radio‐biological evidences. using thresholding values on a_crossing_ and DI, we demonstrated a better proton lung plan determined by DVH constraints learnt from photon world might be worse than the comparing photon plan for lung sparing. In our test group, only about half the cases gEUD(a) with thresholding method yielded the same plan ranking as the conventional DVH evaluation method. To implement proton for lung treatment, careful plan analysis between the proton plan and the photon plan should be carried out prior to clinical practice.

## CONFLICT OF INTEREST

The authors have no conflict of interest to disclose.
